# Primary pleural hydatid cyst: A rare presentation of echinococcosis with diagnostic and therapeutic insights

**DOI:** 10.1016/j.idcr.2025.e02375

**Published:** 2025-09-17

**Authors:** Muhammad Yousaf, Bassem Alhariri, Dore C. Ananthegowda, Maran Paramanandam Anand Paul Kumar, Elmatasam Elawami, Nabil Sherif Mahmood

**Affiliations:** a Hazm Mebaireek Hospital, Hamad Medical Corporation, Doha, Qatar; b College of Medicine, Weill Cornell Medicine-Qatar, Cornell University, Doha, Qatar; c College of Medicine, Qatar University, Doha, Qatar

**Keywords:** Primary pleural hydatid cyst, Echinococcosis, Albendazole, Pleural mass, CT-guided biopsy, Hydatid cyst

## Abstract

**Background:**

Hydatid disease, which is caused by Echinococcus granulosus, mainly impacts the liver and lungs. It is extremely uncommon for the pleura to be primarily affected, and this presents difficulties for diagnosis because it resembles a malignant condition. This report underscores a distinctive instance of an isolated pleural hydatid cyst, focusing on diagnostic challenges, multidisciplinary management, and treatment results.

**Case presentation:**

A Lebanese man aged 60, with no notable exposure history, showed up with an incidental pleural mass. Initial imaging indicated a potential malignancy based on rib erosion and rapid growth. A hydatid cyst was confirmed by a CT-guided biopsy, despite the presence of normal eosinophil counts and negative serology. The MRI ruled out hepatic/pulmonary involvement. Complete resolution, confirmed through serial imaging, was achieved with albendazole monotherapy (400 mg twice daily for five months).

**Discussion:**

This instance highlights how uncommon primary pleural hydatidosis is, as well as the shortcomings of serologic testing. The review of comparative literature shows just 12 documented instances, with our case being the first to exhibit rib erosion without hepatic or pulmonary involvement (Sudan et al., 2025 [2], Soner Gürsoy et al., 2009 [3], Brunetti et al., 2010 [7]). It encompasses radiological, pathological, and therapeutic nuances, promoting biopsy in equivocal cases (Santivanez et al., 2010 [1], Mardani et al., 2017 [6]).

**Conclusion:**

Even though primary pleural hydatid cysts are uncommon, they should be considered in the differential diagnoses of pleural masses, especially in areas where they are endemic. For an accurate diagnosis, histopathology and collaboration across disciplines are essential. For isolated cysts, albendazole monotherapy may be adequate; however, long-term monitoring is crucial (Mardani et al., 2017 [6], Qian, 1988 [9]).

## Highlights

• First reported pleural hydatid cyst causing rib erosion without hepatic involvement.
• Negative Echinococcus serology with normal eosinophils - diagnostic challenge.
• MRI/CT fusion crucial for assessing cyst-chest wall relationship.
• Albendazole monotherapy achieved full radiological resolution.
• Multidisciplinary approach prevented unnecessary thoracotomy

## Introduction

Hydatid disease, caused by the larval stage of *Echinococcus granulosus*, is a zoonotic infection prevalent in regions with close human-animal contact, such as with dogs and livestock. It primarily affects the liver (60–80 %) and lungs (10–30 %), with pleural involvement being exceptionally rare [1]. Primary pleural hydatid cysts, without hepatic or pulmonary parenchymal involvement, are particularly uncommon and pose diagnostic challenges due to their atypical presentation and imaging findings that may mimic other pleural pathologies, such as solitary fibrous tumors or liposarcomas [2]. The rarity of pleural hydatidosis often leads to delayed diagnosis, as it may not be initially considered in the differential diagnosis of pleural lesions [3]. This report presents a rare case of a primary pleural hydatid cyst in a 60-year-old male, highlighting its diagnostic process and management.

## Case summary

A 60-year-old Lebanese man was referred to the pulmonology clinic in January 2023 for evaluation of an incidental pulmonary nodule identified during a cardiac CT angiogram. He worked in the IT department and was a current occasional smoker (< 5 pack years). His medical history included cervical disc prolapse, a benign thyroid nodule, pre-diabetes, and moderate obstructive sleep apnoea. His regular medications included ezetimibe, clopidogrel, esomeprazole, alfuzosin, and rosuvastatin.

The cardiac CT angiogram was performed to evaluate mild exertional dyspnoea, which the patient experienced for over a year following an acute cervical disc prolapse that resulted in reduced mobility. Symptoms gradually resolved over several months. Pulmonary function tests, a six-minute walk test, and cardiology evaluation (echocardiography and stress test) showed no abnormalities. The mild exertional dyspnoea, which resolved spontaneously, was attributed to deconditioning after the cervical disc prolapse. The patient intentionally lost weight (9 kg) due to a recent diagnosis of pre-diabetes. The patient did not report fever or night sweats.

Laboratory Data:

Normal CD4 count (650 cells/µL), IgE (45 IU/mL), CRP (2 mg/L).

Negative Echinococcus serology (ELISA), normal eosinophils (2 %) [5].

Lung Function Tests:

FEV₁: 92 % predicted, FVC: 95 % predicted, DLCO: 88 % predicted.

Physical examination revealed a respiratory rate of 20 breaths per minute, blood pressure of 129/74 mmHg, heart rate of 66 bpm, temperature of 36.2 °C, and oxygen saturation of 99 % on room air. His height was 175 cm, weight 77 kg, and BMI 25.14. There was nothing significant on respiratory examination.

**Fig. 1:**
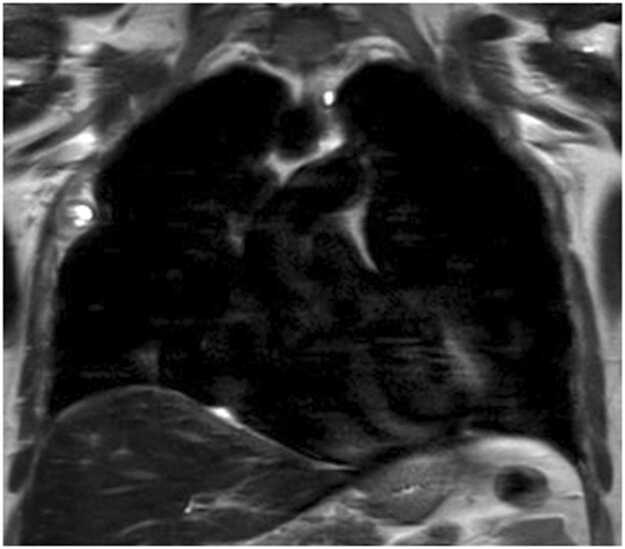
MRI showing primary pleural hydatid cyst: a rare presentation of echinococcosis.

A cardiac CT angiogram in January 2023 detected a 3 mm pulmonary nodule in the left lingula. A high-resolution chest CT in January 2024 identified two nodules: an 8.5 mm calcified nodule in the left upper lobe and a 9.4 × 5 mm nodule in the right upper lobe, along with an incidental 7 × 4 mm well-defined, pleural-based fusiform cystic lesion. A follow-up chest CT in September 2024 showed no growth in the pulmonary nodules, both deemed benign. However, the pleural-based lesion had enlarged significantly (from 7 × 4 mm to 3.1 × 1.7 cm) with adjacent rib erosion. A thoracic MRI confirmed a well-defined fusiform pleural lesion at the right 4th rib level, causing bony erosion (Fig. 1). Differential diagnoses included primary pleural liposarcoma or aggressive solitary fibrous tumor of the pleura.

The case was discussed in the lung cancer multidisciplinary team meeting. A CT-guided biopsy in October 2024 confirmed a hydatid cyst with no malignant features (Fig. 4). Liver involvement was excluded on MRI, and the cyst appeared isolated to the pleura. Laboratory tests, including white cell count, eosinophil count, IgE, CRP, and serology for *Schistosoma* and *Echinococcus*, were normal or negative.

**Fig. 2:**
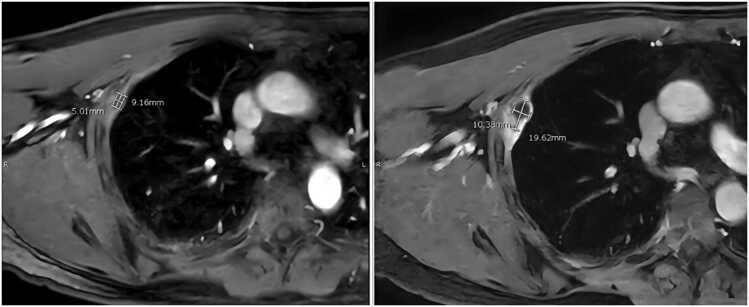
MRI after 8 weeks of treatment revealed significant regression of the pleural hydatid cyst, with the lesion size decreasing from 12 × 10 mm to 7 × 4 mm and resolution of surrounding inflammation.

**Fig. 3:**
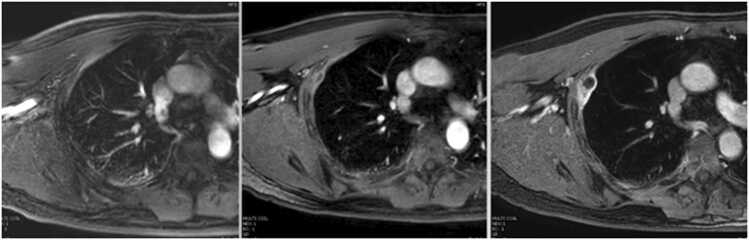
Last MRI confirmed complete resolution of the pleural hydatid cyst.

**Fig. 4:**
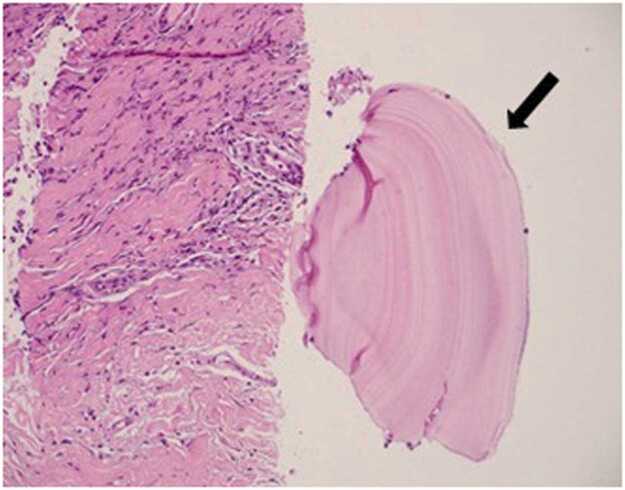
Histopathology slide, hematoxylin and eosin stain shows inflamed connective tissue composed mainly of lymphocytes and eosinophils with areas of fibrosis and adjacent fragments of hydatid cyst wall composed of a laminated acellular eosinophilic membrane (Arrow) [H&E 200 ×].

The patient initiated albendazole 400 mg twice daily for three months. At a four-week follow-up in November 2024, he described a two-week history of intermittent, mild-to-moderate, well-localized, right-sided pleuritic chest pain. No fever, dyspnoea, or weight loss was reported. A repeat thoracic MRI on December 2, 2024, revealed significant regression of the pleural hydatid cyst, with the lesion size decreasing from 12 × 10 mm to 7 × 4 mm and resolution of surrounding inflammation (Fig. 2).

The chest pain subsided within a few weeks. The patient was reassured, and albendazole therapy continued for a total duration of five months. A final MRI, conducted three months after stopping albendazole, confirmed complete resolution of the pleural hydatid cyst (Fig. 3).

## Discussion

Primary pleural hydatid cysts are an extremely rare form of echinococcosis, and our case exhibits several unique characteristics that deserve thorough discussion. This case is especially remarkable due to the occurrence of rib erosion in the absence of hepatic or pulmonary involvement, with only three such documented instances in the literature [2], [7]. Due to the lesion's swift development and damaging alterations to bone structure, this unusual manifestation posed substantial difficulties for diagnosis, with initial indications pointing to malignancy [4].

Multiple factors contributed to the complexity of the diagnostic dilemma in our case. Initially, skepticism about the diagnosis arose from the lack of typical serologic markers, which Santivanez and Garcia [1] report occurs in 20–40 % of confirmed hydatid cases. Secondly, the normal eosinophil count was at odds with the conventional teaching that parasitic infections often come with eosinophilia [5]. The results highlight the shortcomings of depending on lab markers alone for diagnosis, especially in cases that are not typical. Our findings are consistent with the observation by Gürsoy et al. [3] that in cases of this nature, histopathological confirmation is still the gold standard.

Our case's radiological evolution offers significant insights. Although the first cardiac CT angiogram was not optimized for pulmonary evaluation, it incidentally detected a small pleural lesion. The following HRCT allowed for a more detailed description of the lesion's morphology and its connection to neighboring structures [4]. The choice to move forward with the MRI was especially useful for evaluating the integrity of the cyst wall and ruling out mediastinal invasion [7], characteristics that were essential for diagnosis and treatment planning. This imaging sequence illustrates how various modalities serve complementary functions in the assessment of complex pleural pathology.

In this case, careful consideration of several factors was involved in therapeutic decision-making. While surgical excision has traditionally been the mainstay of treatment for hydatid disease [6], we opted for albendazole monotherapy based on several considerations: the small size of the cyst (< 5 cm), its isolated nature, and the absence of complications such as rupture or infection [6], [8]. Our case's remarkable reaction to medical treatment corroborates the suggestion by Brunetti et al. [6] that small, uncomplicated cysts can be treated with medication. The emergence of transient pleuritic pain during treatment underscores the necessity for vigilant observation, as this may indicate early signs of cyst leakage or secondary infection [8].

The biological behavior observed in our case prompts intriguing inquiries regarding the pathogenesis of primary pleural hydatidosis. The lack of hepatic or pulmonary involvement indicates either a hematogenous spread that avoids the hepatic and pulmonary filters, or direct implantation from hidden microscopic foci [7]. The alarming progressive rib erosion was likely caused by chronic pressure effects rather than true invasive behavior, a phenomenon previously described by Aydin et al. [7] in their series of thoracic hydatid cases.

Our case highlights how crucial it is to manage rare thoracic pathologies using a multidisciplinary approach. There were numerous facets of the lung cancer multidisciplinary team discussion that were invaluable, including (1) deciding on the suitable biopsy method, (2) contextualizing the histopathological findings within clinical parameters, and (3) developing a consensus treatment plan [9]. This collaborative method reflects the suggestions of Thameur et al. [9] for handling complex thoracic hydatid disease.

This case provides several lessons that could guide future practice:

Even when typical epidemiological or laboratory findings are absent, primary pleural hydatidosis should be included in the differential diagnosis of enlarging pleural masses [2], [3].

Radiological characteristics indicative of malignancy (such as rib erosion) do not rule out infectious causes [4], [7].

With CT-guided biopsy, it is possible to achieve a conclusive diagnosis and at the same time reduce morbidity [3].

For small, uncomplicated pleural cysts, medical therapy may suffice, while surgery is reserved for more complex cases [6], [8].

We should take into account the long-term consequences of our management approach. Although there was a complete radiologic resolution, it is still unclear what the optimal follow-up duration is. As most recurrences happen within 2 years after treatment [6], this indicates that at least mid-term monitoring is necessary. Furthermore, the possibility of microscopic residual disease existing even when radiologic resolution has occurred supports the need for ongoing vigilance [6], [8].

## Conclusion

This case illustrates a rare presentation of a primary pleural hydatid cyst in a 60-year-old male, initially mistaken for a malignant pleural tumor due to its radiological features and rib erosion. The diagnosis was confirmed through CT-guided biopsy, and treatment with albendazole resulted in complete cyst involution without recurrence. The case underscores the need to include hydatid disease in the differential diagnosis of pleural masses, even when serological or clinical indicators are absent, and highlights the effectiveness of medical treatment for small, isolated pleural hydatid cysts. Long-term surveillance remains crucial to detect potential recurrence.

## CRediT authorship contribution statement

**MAHMOOD Nabil:** Writing – review & editing, Validation. **Elmatasam Elawami:** Writing – review & editing, Writing – original draft. **Kumar Maran:** Writing – review & editing, Writing – original draft. **Ananthegowda Dore:** Writing – review & editing, Writing – original draft. **Al Hariri Bassem:** Writing – review & editing, Writing – original draft, Supervision. **Muhammad Yousaf:** Writing – review & editing, Writing – original draft, Supervision.

## Ethical approval

This case report was subject to review and approval from the Institutional Review Board (IRB) of the Medical Research Center MRC 04-24-798) at Hamad Medical Corporation and is in full conformance with the principles of the "Declaration of Helsinki," Good Clinical Practice (GCP), and within the laws and regulations of MoPH in Qatar. IRB of Hamad Medical Corporation waived the need for informed consent due to the retrospective nature of the study.

## Consent

Written informed consent was obtained from the patient for publication of this case report and any accompanying Figures.

## Declaration of Generative AI and AI-assisted technologies in the writing process

During the preparation of this work, the author(s) used [Grammarly] in order to improve readability and language. After using this tool, the authors reviewed and edited the content as needed and take full responsibility for the content of the published article.

## Funding

This case report was not funded.

## Conflict of interest

The authors have declared that no competing interests exist.

## Declaration of Competing Interest

All authors declare no competing interests.

## Data Availability

The data that support the findings of this study are available in this article; Further enquiries can be directed to the corresponding author.
